# Cutaneous adverse reactions associated with enfortumab vedotin: a pharmacovigilance study based on the FDA adverse event reporting system

**DOI:** 10.3389/fmed.2026.1792256

**Published:** 2026-04-09

**Authors:** Pan Ding, Qinghua Luo, Leihua Cao

**Affiliations:** Department of Urology, Nanchang People's Hospital, Nanchang, China

**Keywords:** cutaneous adverse reactions, data mining, enfortumab vedotin, FAERS, pharmacovigilance

## Abstract

**Background:**

Enfortumab vedotin (EV), a Nectin-4–directed antibody–drug conjugate, improves survival in patients with locally advanced or metastatic urothelial carcinoma (la/mUC) after prior platinum-based chemotherapy and immune checkpoint inhibitors. However, EV-associated cutaneous adverse events (AEs) may be underrecognized in real-world practice.

**Methods:**

We performed a pharmacovigilance study using the U.S. FDA Adverse Event Reporting System (FAERS) database (2020–2025). EV-related cutaneous AE reports were identified and descriptively characterized. Disproportionality analyses were conducted to detect safety signals, and time-to-onset (TTO) was evaluated. Multivariable analyses were used to explore clinical factors associated with cutaneous AEs and death among EV reports.

**Results:**

A total of 1,389 EV-associated cutaneous AE cases were identified. The most frequently reported events were rash, pruritus, and alopecia, alongside severe reactions including Stevens–Johnson syndrome and toxic epidermal necrolysis. We detected 44 positive cutaneous safety signals, including 10 not described in the EV label. Most cutaneous AEs occurred early after treatment initiation and were reported predominantly in elderly male patients. Concomitant prednisolone use was associated with higher reporting of cutaneous AEs and increased mortality, whereas concomitant amlodipine use was associated with lower reporting of cutaneous AEs.

**Conclusion:**

Real-world FAERS data indicate that enfortumab vedotin is associated with a broad and evolving spectrum of cutaneous adverse events, including early-onset and potentially life-threatening reactions. We identified 10 previously unlabeled safety signals, and most events occurred within the first 2 weeks after treatment initiation. The proposed clinical priority framework may help support risk stratification and pharmacovigilance monitoring. Although causality cannot be established using spontaneous reporting data, these findings highlight the importance of early dermatologic surveillance and individualized safety management during EV therapy.

## Introduction

Urothelial carcinoma (UC) is a malignancy originating from the urothelial lining of the renal pelvis, ureters, bladder, and urethra. Approximately 90% of UC cases arise in the bladder, making it the 10th most common cancer worldwide (1, 2). According to the 2020 GLOBOCAN statistics, there were an estimated 573,000 new cases of bladder cancer and 213,000 related deaths globally (3). At diagnosis, around 70%−75% of patients present with non-muscle-invasive bladder cancer (NMIBC). Although early-stage bladder cancer is generally responsive to surgical treatment, the disease is characterized by a high recurrence rate (31%−78%) and a risk of progression (4, 5). Approximately 5% of cases are initially diagnosed as locally advanced or metastatic disease (la/mUC), which significantly worsens the prognosis (6). For patients with metastatic disease, the five-year overall survival rate remains below 5% (7). Thus, the development of effective and innovative therapeutic strategies for bladder cancer, particularly in its advanced stages, remains a major focus of clinical and translational research.

For decades, cisplatin-based chemotherapy has served as the standard of care for patients with la/mUC, offering a median overall survival (mOS) of 14–15 months (8). However, nearly half of these patients are ineligible for cisplatin due to renal insufficiency or comorbidities (9). Carboplatin-based regimens have demonstrated inferior efficacy. In the era of precision medicine, immune checkpoint inhibitors have improved outcomes for a subset of patients, but response rates remain modest (20%−30%), and resistance frequently develops (10). The advent of antibody–drug conjugates (ADCs) has revolutionized the therapeutic landscape for advanced bladder cancer. Enfortumab vedotin (EV), the first ADC targeting Nectin-4, selectively delivers cytotoxic agents to tumor cells via an antibody-mediated mechanism. It received accelerated FDA approval in December 2019 for the treatment of la/mUC (11). Clinical studies have shown that EV significantly improves objective response rates (ORR) and prolongs progression-free survival (PFS) in patients with metastatic bladder cancer who have failed treatment with prior PD-1/PD-L1 inhibitors (12, 13). Compared with conventional chemotherapy, EV further extends overall survival (OS) (14), making it a preferred treatment option for patients treatment with refractory to standard chemotherapy and immunotherapy.

Despite the therapeutic promise of EV in advanced or metastatic UC, its associated adverse events warrant careful attention, with cutaneous reactions among the most frequently reported (15). In the EV-301 trial, treatment-related skin AEs of any grade occurred in 47% of patients receiving EV, compared to 15.8% in the chemotherapy arm. Notably, Grade ≥3 cutaneous adverse events—classified as adverse events of special interest (AESIs)—occurred in 14.5% of the EV group vs. 0.7% of the chemotherapy group. These toxicities are likely due to the expression of Nectin-4 on normal epidermal cells, eccrine glands, and hair follicles, leading to off-target toxicities (16). Due to the strict inclusion criteria in clinical trials, study populations may not fully reflect the diversity of real-world patients, limiting the generalizability of safety findings. In contrast, real-world data capture a broader patient spectrum and is crucial for identifying rare or unexpected AEs that may not surface in controlled trial settings (17). Since EV's approval, there have been increasing reports of serious blistering skin reactions and even fatal dermatologic events such as Stevens–Johnson Syndrome (SJS) and toxic epidermal Necrolysis (TEN), prompting a boxed warning (18–20). Therefore, it is particularly important to conduct in-depth investigation and close monitoring of Enfortumab vedotin–induced cutaneous toxicity.

The U.S. FDA Adverse Event Reporting System (FAERS) is a publicly accessible database used to monitor post-marketing drug safety and represents one of the largest pharmacovigilance resources globally (21). Although Hui Yang et al. previously investigated EV-associated skin toxicity using FAERS data (22), the growing clinical use of EV and the increasing volume of AE reports highlight the need for updated and comprehensive analyses. In this study, we conducted a detailed pharmacovigilance investigation using FAERS data from Q1 2020 to Q1 2025 to characterize the spectrum and frequency of EV-related cutaneous adverse events. We further explored these events through stratified analyses, clinical prioritization of AE signals, time-to-onset (TTO) distribution modeling, and evaluation of serious clinical outcomes. Our findings aim to inform early clinical warning systems, support individualized treatment strategies, and enhance risk management in the use of EV, providing robust real-world evidence to guide safe and effective treatment in la/mUC.

## Method

### Study design and data source

This study adopted an observational, retrospective disproportionality analysis approach, a widely used pharmacovigilance method for signal detection in spontaneous reporting systems. The objective was to quantify the disproportionality between enfortumab vedotin and cutaneous adverse events (AEs) and thereby identify potential safety signals. Data were obtained from the FDA Adverse Event Reporting System (FAERS), publicly accessible in compressed ASCII or XML formats from the FAERS Quarterly Data Extract Files (https://fis.fda.gov/extensions/fpd-qde-faers/fpd-qde-faers.html). The study included all FAERS reports involving enfortumab vedotin from Q1 2020 through Q1 2025.

### Data extraction and descriptive analysis

FAERS consists of seven datasets: demographic and administrative data (DEMO), drug information (DRUG), adverse events (REAC), patient outcomes (OUTC), report sources (RPSR), therapy start/end dates (THER), and indications (INDI). All data were imported into the R software (version 4.3.1). To ensure accuracy, duplicate records were removed following FDA recommendations. Specifically, records were sorted by CASEID, FDA_DT, and PRIMARYID; for duplicate CASEIDs, the record with the most recent FDA_DT was retained. If both CASEID and FDA_DT were identical, the record with the highest PRIMARYID was selected (23). This process ensured that each patient was represented by a single unique report.

Cases were identified by searching for “Enfortumab vedotin” in both the generic name and PROD_AI fields, as well as its trade name, “Padcev,” in the DRUG file. Only records where enfortumab vedotin was marked as the primary suspect drug (“PS”) were included. Combination therapy was defined as reports involving enfortumab vedotin (as PS) alongside other drugs labeled as secondary suspect (“SS”), concomitant (“C”), or interacting (“I”). For duplicate CASEIDs, the record with the highest PRIMARYID was manually selected.

**Figure 1:**
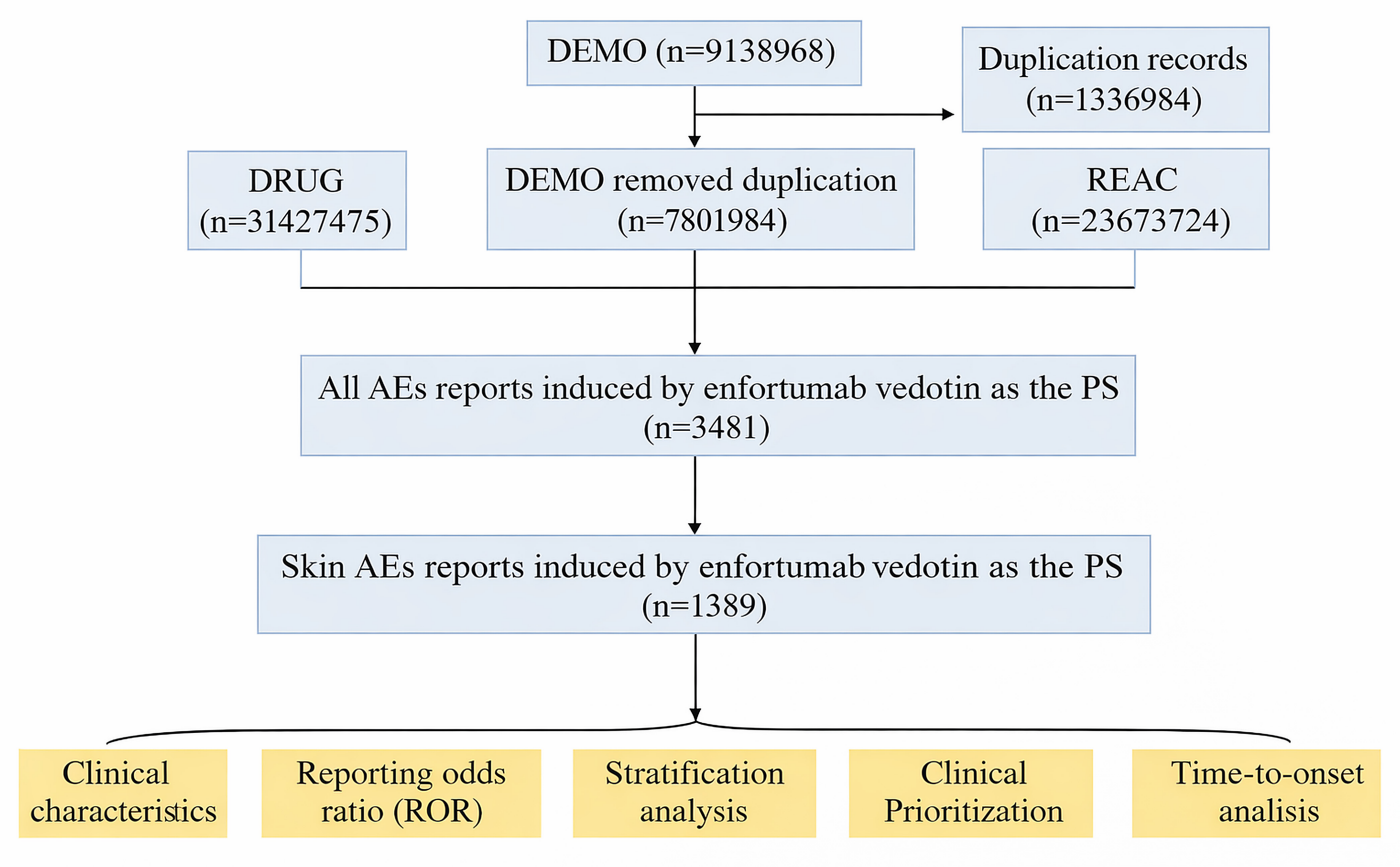
Flowchart of the study. Study flowchart outlining the identification and selection of reports involving enfortumab vedotin from the FAERS database (Q1 2020 - Q1 2025).

Adverse events were coded using MedDRA Preferred Terms (PTs), classified under the 27 System Organ Classes (SOCs). In this study, MedDRA version 27.2 was used, and all PTs under the “Skin and Subcutaneous Tissue Disorders” SOC (code: 10040785) were included. Clinical characteristics such as sex, weight, age, reporter type, country, outcome, and report date were analyzed descriptively. Serious outcomes included death, disability, hospitalization, or other medically significant conditions. Since one case could report multiple outcomes, the total count may exceed the number of unique reports. A detailed flowchart of the data extraction, deduplication, and analysis is presented in Figure 1.

## Statistical analysis

Disproportionality analysis was conducted using the Reporting Odds Ratio (ROR), a well-established indicator in pharmacovigilance-based signal detection based on 2 × 2 contingency tables (Supplementary Table S1) (24). ROR compares the odds of reporting a specific AE for Enfortumab vedotin vs. all other drugs. A signal was considered positive if the lower limit of the 95% confidence interval (ROR025) exceeded 1, and the number of AE reports was ≥3. The strength of signals under the “Skin and Subcutaneous Tissue Disorders” SOC was also evaluated.

We compared serious and non-serious reports to assess the clinical significance of each signal and identify risk factors (e.g., sex, age, weight). Categorical variables were analyzed using Pearson's chi-square test (χ^2^). All analyses were conducted using the R software (version 3.2.1), with a significance threshold of *p* < 0.05. Subgroup analyses were performed by sex (male vs. female), age (18–64 vs. ≥65 years), weight (< 80 kg, 80–100 kg, >100 kg), and reporter type (healthcare professionals vs. non-healthcare professionals).Because enfortumab vedotin dosing is capped at 125 mg and dose information is frequently missing in FAERS, weight-normalized dosing (mg/kg) could not be reliably calculated; therefore, body weight was analyzed as a categorical variable.

### Clinical priority ranking of safety signals

A semi-quantitative scoring system was used to prioritize safety signals based on four criteria: classification as designated medical events or important medical events, proportion of AE reports, signal stability, and proportion of fatal outcomes. AEs were categorized into three priority levels: low (score 0–2), moderate (score 3–5), and high (score 6–8) (25). Signals with moderate or high priority were considered clinically significant and warranted close monitoring.

### Time-to-onset (TTO) analysis

TTO was defined as the interval between the therapy start date (START_DT in the THER file) and the event date (EVENT_DT in the DEMO file). Reports with implausible dates (e.g., EVENT_DT earlier than START_DT) were excluded. TTO distributions were described using the median, interquartile range (IQR), and Weibull shape parameter (WSP). The Weibull distribution is characterized by scale (α) and shape (β) parameters: β < 1 (95% CI < 1): early failure trend; β ≈ 1 (95% CI includes 1): random failure; β >1 (95% CI excludes 1): wear-out failure trend. We calculated TTO medians and WSPs for AEs categorized as high-, moderate-, or low-priority clinical priority to explore time-dependent risk patterns.

### Logistic regression analysis

We identified the top 10 most frequently co-administered drugs with enfortumab vedotin in the deduplicated DRUG table. Variables such as age and sex (from the DEMO file) were extracted as independent variables. Univariate and multivariate logistic regression analyses were conducted to examine associations between these factors and the occurrence of cutaneous AEs. The same set of variables was used to model the risk of fatal outcomes as the dependent variable. All analyses were performed using the R (version 3.2.1) and Minitab Statistical Software (v20.0; Minitab LLC, State College, PA, USA).Only variables reliably available in FAERS (age, sex, and the most frequently co-reported concomitant medications) were included in multivariable models, while other clinically relevant confounders could not be adjusted due to data limitations.

## Results

### Descriptive analysis

A total of 9,138,968 adverse event (AE) reports were retrieved from the FAERS database. After deduplication, 3,481 reports identified enfortumab vedotin as the primary suspect (PS), among which 1,389 involved cutaneous adverse events. The clinical characteristics of these cases are summarized in Table 1. Among patients with EV-induced cutaneous AEs, the majority were male (1,000 cases, 71.99%). Of the 297 cases with available weight data, 2.23% weighed more than 100 kg, 2.88% weighed between 80–100 kg, and 16.27% weighed less than 80 kg. Elderly patients (≥65 years) accounted for the largest proportion (685 cases, 49.32%), followed by middle-aged adults (198 cases, 14.25%) and younger patients (183 cases, 13.17%). Most reports were submitted by healthcare professionals (990 cases, 71.27%). Geographically, the majority of reports originated from Japan (592 cases, 42.62%) and the United States (510 cases, 36.72%). Serious outcomes were reported in 1,126 cases (81.07%), including 235 deaths (16.92%), 8 disabilities (0.58%), 369 hospitalizations (26.57%), 46 life-threatening events (3.31%), and 468 other serious outcomes (33.69%). Reports of cutaneous AEs associated with enfortumab vedotin have shown a steadily increasing trend since its market approval.

**Table 1:** Clinical characteristics of patients with cutaneous adverse events related to enfortumab vedotin treatment.

Characteristics	Skin AEs	Overall
	*N* = (1,389)	*n* = (3,481)
Sex		
Female	347 (24.98%)	787 (22.61%)
Male	1,000 (71.99%)	2,559 (73.51%)
Missing	42 (3.02%)	135 (3.88%)
WT		
< 80kg	226 (16.27%)	491 (14.10%)
80 100 kg	40 (2.88%)	74 (2.13%)
>100kg	31 (2.23%)	57 (1.64%)
Missing	1,092 (78.62%)	2,859 (82.13%)
Age		
< 18	183 (13.17%)	528 (15.17%)
18 65	198 (14.25%)	518 (14.88%)
>65	685 (49.32%)	1,511 (43.41%)
Missing	323 (23.25%)	924 (26.54%)
Reporter type		
Consumer	390 (28.08%)	964 (27.69%)
Health professional	214 (15.41%)	531 (15.25%)
Physician	660 (47.52%)	1,693 (48.64%)
Pharmacist	116 (8.35%)	275 (7.90%)
Lawyer	1 (0.07%)	1 (0.03%)
Missing	8 (0.58%)	17 (0.49%)
Country (top five)		
Japan	592 (42.62%)	1,410 (40.51%)
United States	510 (36.72%)	1,239 (35.59%)
France	90 (6.48%)	172 (4.94%)
Canda	38 (2.74%)	103 (3.73%)
Germany	18 (1.30%)	69 (1.98%)
Others	141 (10.15%)	488 (14.02%)
Outcome		
Serious outcome	1,126 (81.07%)	2,950 (84.75%)
Death	235 (16.92%)	681 (19.56%)
Disability	8 (0.58%)	14 (0.40%)
Hospitalization	369 (26.57%)	869 (24.96%)
Life-threatening	46 (3.31%)	105 (3.02%)
Other	468 (33.69%)	1,281 (36.80%)
Non-serious outcome	263 (18.93%)	531 (15.25%)
Reporting year		
2025 Q1	138 (9.94%)	355 (10.20%)
2024	475 (34.20%)	1,084 (31.14%)
2023	387 (27.86%)	952 (27.35%)
2022	176 (12.67%)	536 (15.40%)
2021	130 (8.35%)	321 (9.22%)
2020	83 (5.98%)	233 (6.69%)

AEs, adverse events; *n*, number of cases.

### Signal detection analysis

**Figure 2:**
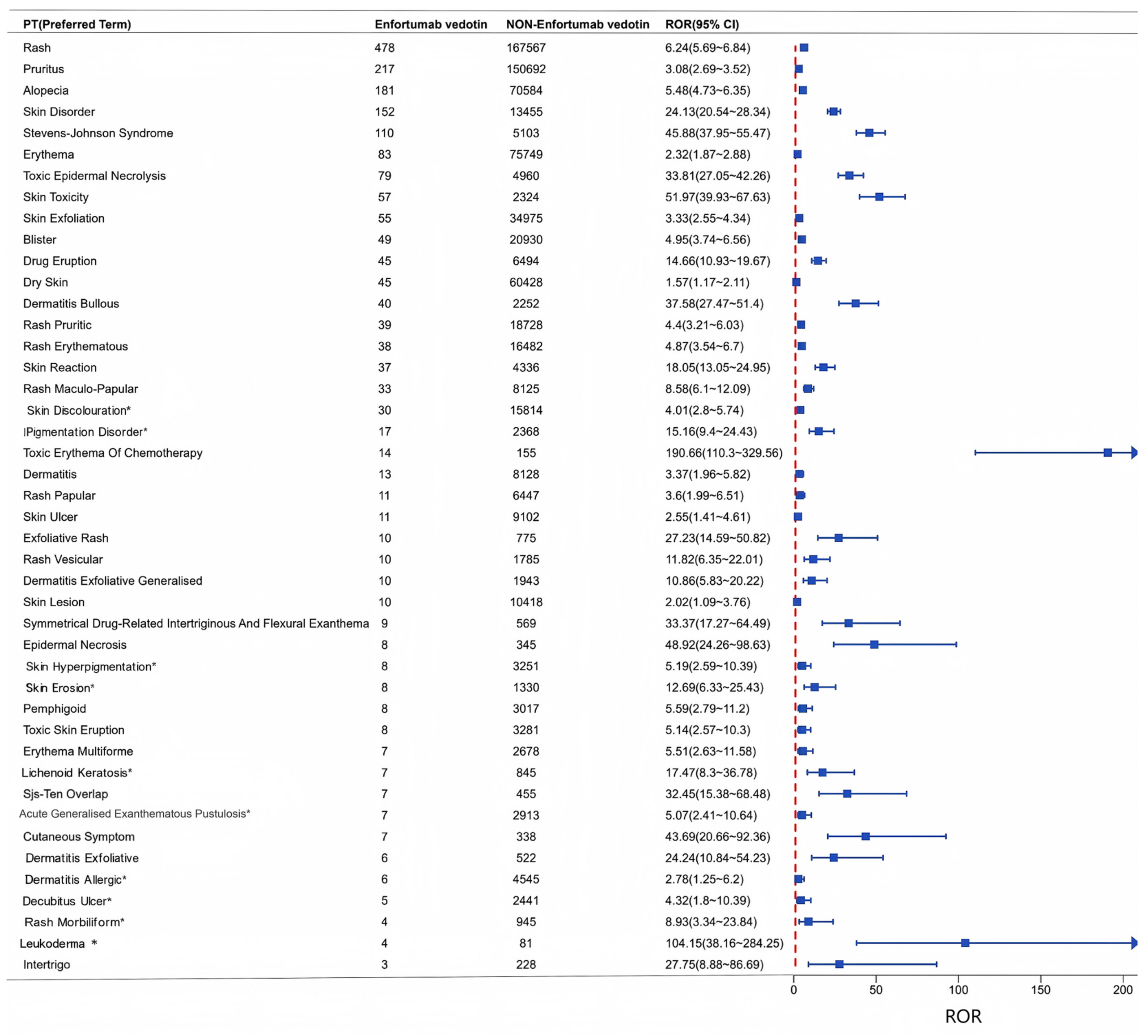
Disproportionality analysis of cutaneous adverse events associated with enfortumab vedotin. Term-level signals, ranked by descending reporting odds ratio (ROR). Blue squares represent the ROR point estimate, with horizontal lines indicating the 95% confidence interval (CI). The vertical dashed red line marks the threshold of ROR = 1. Rash, pruritus, and alopecia were the most frequently reported events, while the strongest signal was observed for chemotherapy-related erythema (ROR 190.66, 95% CI 110.3–329.56). Ten signals, such as skin discolouration and pigmentation disorder, were not listed in the drug label. Data are from the FAERS database (Q1 2020 to Q1 2025).

Using the ROR method, 44 positive PT-level signals were identified for enfortumab vedotin (EV)-associated cutaneous AEs (Figure 2). The most frequently reported reactions included rash (*n* = 478), pruritus (*n* = 217), alopecia (*n* = 181), skin lesions (*n* = 152), Stevens–Johnson syndrome (*n* = 110), erythema (*n* = 83), toxic epidermal necrolysis (*n* = 79), skin toxicity (*n* = 57), exfoliative dermatitis (*n* = 55), and blisters (*n* = 49). These 44 AEs showed stronger signal intensities compared to those associated with non-enfortumab vedotin drugs, with ROR025 values ranged from 1.57 (dry skin) to 190.66 (chemotherapy-related erythema). At the SOC level, “Skin and Subcutaneous Tissue Disorders” had the highest ROR of 4.02 (95% CI: 3.83–4.21), compared to a ROR of 0.25 (95% CI: 0.24–0.26) for non-skin-related AEs (Supplementary Table S2). Notably, 10 novel signals not listed in the drug label were identified: skin discolouration, pigmentation disorder, skin hyperpigmentation, skin erosion, lichenoid keratosis, acute generalized exanthematous pustulosis, allergic dermatitis, decubitus ulcer, morbilliform rash, and leukoderma.

### Serious vs. non-serious cases

As shown in Supplementary Table S3, statistically significant differences (*p* < 0.05) in age, sex, and weight were observed between serious and non-serious cutaneous AE cases. Six AEs were significantly more likely to be reported as serious events: alopecia, erythematous rash, macular rash, skin hyperpigmentation, skin reaction, and toxic epidermal necrolysis. Conversely, 34 AEs were more frequently reported as non-serious events, including blistering, cutaneous symptoms, dermatitis, allergic dermatitis, drug eruption, and dry skin.

### Stratified analyses

To improve the robustness of the findings, four stratified analysis strategies were applied. As shown in Table 2, ROR values remained statistically significant (lower limit of 95% CI >1) across all subgroups stratified by sex, age, body weight, and reporter type, confirming a strong association between enfortumab vedotin and cutaneous disorders.

**Table 2:**
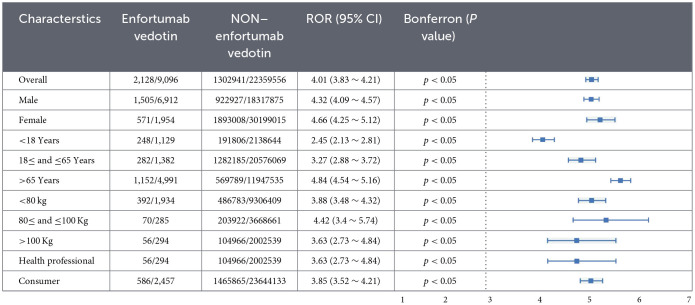
Subgroup analysis of cutaneous toxicities associated with enfortumab vedotin.

CI, confidence interval; *N*, number of cases of total; AEs, associated with the target drug; ROR, reporting odds ratio; *n*, number of cases with suspected; AEs, associated with the target drug.

### Clinical priority ranking

As detailed in Supplementary Table S4, among the 44 EV-associated cutaneous AEs, 25 were categorized as low clinical priority, 18 as moderate, and 1 as high. The only high-priority AE was toxic epidermal necrolysis, with a composite score of 6.

### Time-to-onset analysis

Supplementary Table S5 presents TTO and Weibull shape parameter (WSP) data for high-, moderate-, and low-priority signals. The median TTOs were 13 days (IQR: 8.75–20) for high-priority, 11 days (IQR: 7–20) for moderate-priority, and 13 days (IQR: 7–22) for low-priority events. Interestingly, moderate-priority events tended to occur earlier than both high- and low-priority AEs. WSP analysis indicated that all β values and their 95% confidence intervals were below 1, suggesting an early failure pattern for these AEs.

### Logistic regression analysis

As shown in Tables 3, 4, Univariate logistic regression revealed that female sex was a protective factor, while concomitant use of prednisolone was a significant risk factor for EV-induced cutaneous AEs. In multivariate logistic regression, amlodipine co-administration was independently associated with a protective effect, whereas prednisolone remained a significant risk factor. Further univariate analysis of fatal outcomes demonstrated that prednisolone co-administration was significantly associated with an increased risk of death.

**Table 3:**
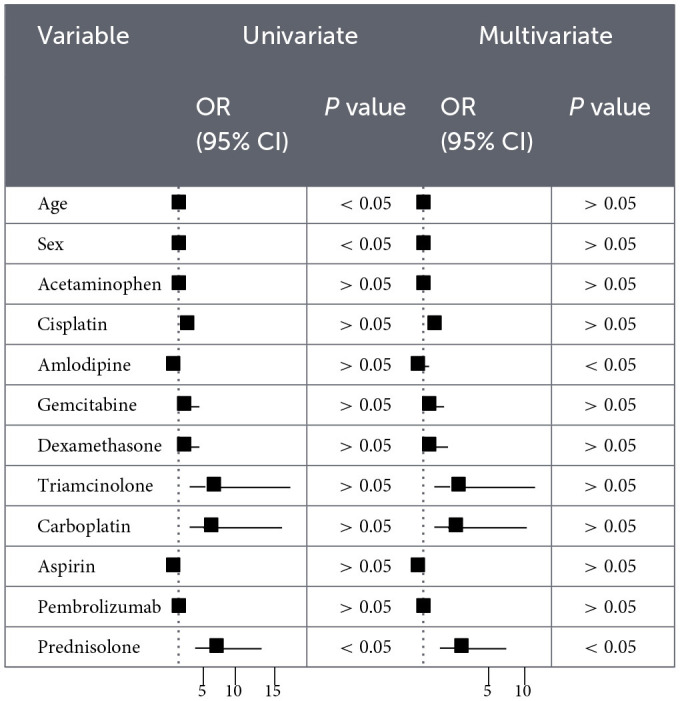
Logistic regression analysis (univariate and multivariate) of enfortumab vedotin-associated skin adverse events.

CI, confidence interval; OR, odds ratio.

**Table 4:**
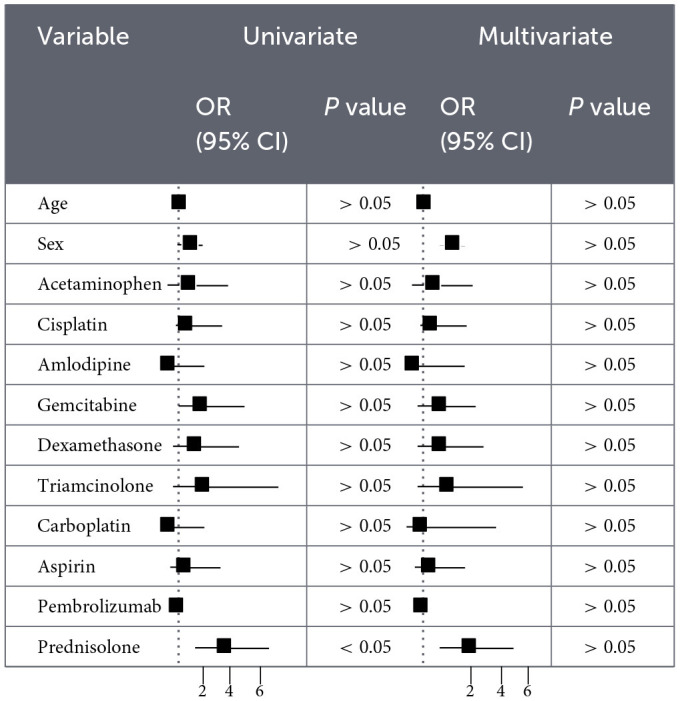
Logistic regression analysis (univariate and multivariate) of enfortumab vedotin-related mortality.

CI, confidence interval; OR, odds ratio.

## Discussion

This study provides the most up-to-date real-world evidence regarding Enfortumab vedotin (EV)-associated cutaneous adverse events (AEs) using post-marketing data from the FAERS database. Beyond characterizing the clinical features of EV-related skin toxicities, we incorporated stratified analyses, clinical priority scoring, severity assessment, and logistic regression modeling to enhance signal interpretation and risk identification.

Compared with the pharmacovigilance study conducted by Hui Yang et al. (22), which analyzed FAERS data from 2019 to 2021 and identified 212 cases of EV-associated cutaneous toxicities, our study substantially extends the surveillance window through the first quarter of 2025 and captures a markedly larger number of reports (*n* = 1,389), reflecting the increasing real-world utilization of EV. Notably, we also identified 10 cutaneous adverse event signals not currently described in the product labeling. Although causality cannot be established within a spontaneous reporting system, these findings suggest that the evolving safety profile of EV warrants continued pharmacovigilance surveillance.

Descriptive analysis (Table 1) revealed a notable sex disparity, with male patients constituting the majority of cutaneous AE reports. This finding aligns with the higher incidence of bladder cancer in men (26). The majority of EV-related cutaneous AEs occurred in older adults (≥65 years), consistent with the epidemiology of urothelial carcinoma (27). Patients weighing less than 80 kg were more likely to experience skin toxicities than those over 80 kg. This may be attributed to higher drug exposure per kilogram of body weight in lighter individuals, whereas dose capping at 125 mg for patients weighing ≥100 kg may mitigate toxicity (20, 28). However, due to incomplete dosing information in the FAERS database, precise evaluation of weight-normalized drug exposure is not feasible; therefore, this finding should be interpreted with caution. To enhance the reliability of our findings, stratified analyses demonstrated consistent patterns across sex, age, and weight subgroups (Table 2). Geographically, most reports originated from Japan and the United States—regions characterized by a high proportion of older adults and strong clinical adoption due to their involvement in pivotal EV trials (27, 28, 47), respectively. Serious outcomes accounted for 81.07% of all cutaneous AE reports, and the number of skin toxicity reports has markedly increased over time, emphasizing the urgent need for enhanced pharmacovigilance efforts.

Through disproportionality analysis, we identified 44 positive PT-level cutaneous AE signals associated with EV (Figure 2), with the most frequent being rash (*n* = 478), pruritus (*n* = 217), alopecia (*n* = 181), skin lesions (*n* = 152), Stevens–Johnson syndrome (SJS, *n* = 110), and erythema (*n* = 83)—findings that are largely consistent with previous clinical studies (29). Mechanistically, EV exerts its antitumor effect by targeting Nectin-4 on cancer cells, enabling the delivery of monomethyl auristatin E (MMAE) into the cytoplasm following lysosomal cleavage. However, this same mechanism may also affect normal tissues expressing Nectin-4, such as keratinocytes, sweat glands, and hair follicles, leading to off-target skin toxicity (30, 31, 46). Toxic erythema of chemotherapy demonstrated the strongest signal (ROR025 = 190.66), followed by leukoderma (ROR025 = 104.15), a reaction not listed in the current label and therefore warranting long-term monitoring. It is also important to note that toxic erythema of chemotherapy (TEC) may overlap clinically with other severe cutaneous adverse reactions in patients receiving anticancer therapies. Severe TEC can present with painful erythematous, bullous, or desquamative lesions and may mimic Stevens–Johnson syndrome/toxic epidermal necrolysis (SJS/TEN) (32). In the setting of enfortumab vedotin treatment, some reactions initially considered TEN have been reported to show histopathologic features more consistent with TEC, suggesting possible diagnostic overlap between these entities (33). Given the lack of detailed clinical, dermatopathologic, and longitudinal information in FAERS, diagnostic misclassification between TEC and TEN cannot be excluded in spontaneous reports. Therefore, the very strong disproportionality signal observed for TEC in our study should be interpreted in the context of possible phenotypic overlap with TEN and related severe exfoliative reactions. Among these signals, the most life-threatening was Stevens–Johnson syndrome/toxic epidermal necrolysis (SJS/TEN), known for its high mortality rate (34). The underlying mechanism may involve type IV delayed hypersensitivity reactions mediated by CD8+ cytotoxic T cells, cytokines, and chemokines (35), and potentially T-helper 17 (Th17)-driven inflammation in skin lesions (36). Further experimental and clinical research are warranted to confirm these mechanisms.

Importantly, we identified multiple skin AEs not currently listed in the Padcev label, including skin discolouration, pigmentation disorder, skin hyperpigmentation, skin erosion, lichenoid keratosis, acute generalized exanthematous pustulosis (AGEP), allergic dermatitis, decubitus ulcer, morbilliform rash, and leukoderma. These findings suggest that the cutaneous toxicity spectrum of EV may be broader than previously recognized, underscoring the need for ongoing pharmacovigilance and evidence-based updates to the product labeling.

Existing studies report that 14% of EV-treated patients develop grade 3–4 skin AEs requiring dose modification or treatment discontinuation (20). Our analysis showed that serious AEs accounted for over 81% of cutaneous events (Table 2), with age, sex, and body weight significantly influencing severity. This highlights the need for heightened monitoring in older, male, and underweight patients to reduce the risk of treatment interruption and improve outcomes. Interestingly, certain AEs—such as blistering, cutaneous symptoms, dermatitis, drug eruptions, and dry skin—differed significantly between serious and non-serious cases, suggesting their potential role as early clinical predictors. Future studies should investigate the mechanistic links between these demographic factors and skin toxicities, and develop predictive risk models to support personalized treatment strategies.

We also developed a novel clinical priority scoring system that integrates signal strength, clinical impact, and robustness. This multidimensional evaluation provides clinically actionable insights for healthcare providers. Toxic epidermal necrolysis (TEN) received the highest score (6), aligning with its inclusion in the Boxed Warning and its known fatality rate of up to 40% (37, 38). Moderate-priority events included rash, pruritus, alopecia, SJS, and various exfoliative and bullous disorders—most of which are already acknowledged in the Padcev label, thereby supporting the validity of our scoring model. Prospective studies should further validate this tool and explore the contribution of individual risk factors to high-priority AEs, in order to optimize clinical decision-making and AE management. However, this scoring framework is semi-quantitative and involves subjective weighting of multiple criteria. It was developed as an exploratory prioritization tool, and further validation in independent datasets is required to confirm its generalizability and clinical applicability.

The Weibull shape parameter was employed to model AE time-to-onset (TTO) patterns and guide clinical risk assessment. The median TTOs for high-, moderate-, and low-priority signals were 13, 11, and 13 days, respectively—findings that align with those reported in clinical trials (13). All disproportional signals displayed early failure characteristics (β < 1), indicating that most cutaneous AEs occur within the first 2 weeks of EV initiation. This finding emphasizes the importance of close skin monitoring, particularly during the early treatment phase, and supports current recommendations for early risk mitigation, such as identifying high-risk patients, reinforcing skin care practices, and implementing timely dose modifications.

We further explored potential risk patterns using logistic regression analyses. In univariate models, female sex was associated with lower reporting of cutaneous AEs, whereas concomitant prednisolone use was associated with higher reporting. In multivariable analyses, amlodipine use showed an exploratory association with lower reporting of cutaneous AEs, while prednisolone remained associated with increased reporting. These findings should be interpreted cautiously, as residual confounding and confounding by indication cannot be excluded. The observed association involving amlodipine may be related to its reported immunomodulatory or skin barrier–supportive properties; however, this remains speculative and requires further investigation (39–41). Similarly, although prednisolone is commonly used to manage inflammatory reactions (42), its association with increased AE reporting may reflect underlying disease severity, treatment indication, or other unmeasured clinical factors rather than a direct pharmacologic effect (43–45). Regarding fatal outcomes, univariate analysis identified prednisolone co-administration as a risk factor. However, this association was no longer statistically significant in the multivariate model, suggesting potential confounding by disease severity, cancer stage, or comedications (49). As corticosteroids are often prescribed in severe cases, they may act as proxies for underlying disease severity rather than independent predictors. Future prospective studies using real-world data should further explore these associations and elucidate their underlying biological mechanisms.

This pharmacovigilance study, based on the FDA Adverse Event Reporting System (FAERS), has several inherent limitations. First, as a spontaneous reporting system, FAERS is subject to under-reporting, reporting bias, variability in report quality, and incomplete clinical information. Second, the database lacks detailed patient-level data, including accurate dosing, treatment duration, comorbidities, and cumulative drug exposure, which limits the ability to adjust for potential confounding factors. Third, the absence of denominator data precludes estimation of true incidence rates. Finally, although disproportionality analyses such as the ROR method can identify statistical associations, they cannot establish causal relationships. Therefore, the detected signals should be interpreted as hypothesis-generating safety alerts rather than confirmed causal links.

## Conclusion

This real-world pharmacovigilance study systematically investigated Enfortumab vedotin–associated cutaneous adverse events using the reporting odds ratio (ROR) method. A total of 44 positive skin-related signals were identified, including 10 novel cutaneous adverse events not previously documented in the current product labeling. Patient age, sex, and body weight were significantly associated with the severity of skin toxicities, with older, male, and low-weight individuals being more susceptible to severe reactions. By constructing a clinical priority scoring system, we stratified the 44 signals into one high-priority, 18 moderate-priority, and 25 low-priority events. The median time-to-onset (TTO) for all signals was within 2 weeks—suggesting a predominant early-onset pattern and underscoring the importance of vigilant monitoring during the initial phase of treatment. Further regression analysis revealed that co-administration with amlodipine or prednisolone was independently associated with EV-induced cutaneous AEs. These findings offer valuable insights for developing personalized medication strategies and targeted risk mitigation approaches. Although based on a large-scale spontaneous reporting system, the study highlights emerging safety concerns surrounding EV that warrant further validation through prospective clinical trials and robust real-world evidence to refine the safety profile and guide clinical management.

## Data Availability

The datasets presented in this study can be found in online repositories. The names of the repository/repositories and accession number(s) can be found in the article/Supplementary material.
