# Supplementary material for: Cutaneous adverse reactions associated with enfortumab vedotin: a pharmacovigilance study based on the FDA adverse event reporting system
Source: Front Med (Lausanne). 2026 Apr 9;13:1792256. doi: 10.3389/fmed.2026.1792256 (PMC13102616; doi:10.3389/fmed.2026.1792256)
Supplement: Supplementary file 1 [file Supplementary_file_1.docx]

Supplementary Material

# Supplementary Data

Supplementary Material should be uploaded separately on submission. Please include any supplementary data, figures and/or tables.

Supplementary material is not typeset so please ensure that all information is clearly presented, the appropriate caption is included in the file and not in the manuscript, and that the style conforms to the rest of the article.

# Supplementary Figures and Tables

For more information on Supplementary Material and for details on the different file types accepted, please see [here](https://www.frontiersin.org/guidelines/author-guidelines#supplementary-material).

## Supplementary Figures

**
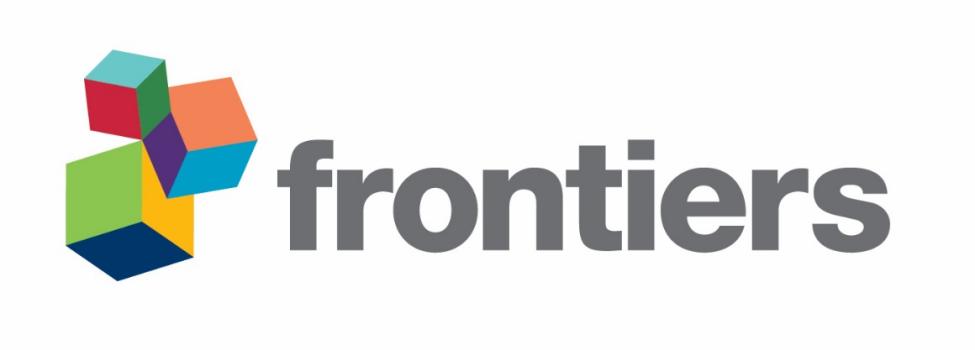
**

**Supplementary Figure 1.** The figure legends are required to have the same font as the main text, 12 point normal Times New Roman, single spaced. Please use a single paragraph for each legend and prepare the figures keeping in mind the PDF layout.

**Supplementary Table S1**. Calculation of reporting odds ratio (ROR).

|  | Reports with target AE | Reports without target AE |
| --- | --- | --- |
| Reports with enfortumab vedotin | a | b |
| Reports without enfortumab vedotin | c | d |

a, number of reports containing both the target drug (enfortumab vedotin) and target AE; b, number of reports containing other AEs of the target drug; c, number of reports containing the target AE of other drugs; d, number of reports containing other drugs and other AEs.

AEs, Adverse Events; ROR, Reporting Odds Ratio; CI, confidence interval.

The calculation formulas are shown below:

1. ROR=ad/b/c
2. 95%CI=e^ln(ROR)±1.96(1/a+1/b+1/c+1/d)^0.5^

**Supplementary Table S2.** Signal strength of reports of enfortumab vedotin at the System Organ Class (SOC) level in the FAERS database

| SOC name | n | ROR(95%Cl) |
| --- | --- | --- |
| Renal And Urinary Disorders | 293 | 1.48 ( 1.31 - 1.66 ) |
| Blood And Lymphatic System Disorders | 572 | 3.07 ( 2.82 - 3.34 ) |
| Respiratory, Thoracic And Mediastinal Disorders | 392 | 0.76 ( 0.69 - 0.85 ) |
| Cardiac Disorders | 126 | 0.58 ( 0.48 - 0.69 ) |
| Eye Disorders | 232 | 1.06 ( 0.93 - 1.2 ) |
| Gastrointestinal Disorders | 968 | 1.1 ( 1.03 - 1.18 ) |
| Nervous System Disorders | 1000 | 1.26 ( 1.18 - 1.35 ) |
| Infections And Infestations | 611 | 0.95 ( 0.88 - 1.04 ) |
| Hepatobiliary Disorders | 241 | 2.59 ( 2.28 - 2.95 ) |
| Immune System Disorders | 33 | 0.25 ( 0.18 - 0.36 ) |
| Investigations | 706 | 1.09 ( 1.01 - 1.18 ) |
| Skin And Subcutaneous Tissue Disorders | 2127 | 4.02 ( 3.83 - 4.21 ) |
| Neoplasms Benign, Malignant And Unspecified (Incl Cysts And Polyps) | 628 | 1.53 ( 1.41 - 1.66 ) |
| General Disorders And Administration Site Conditions | 1422 | 0.68 ( 0.64 - 0.71 ) |
| Metabolism And Nutrition Disorders | 761 | 3.73 ( 3.46 - 4.01 ) |
| Psychiatric Disorders | 110 | 0.18 ( 0.15 - 0.21 ) |
| Injury, Poisoning And Procedural Complications | 583 | 0.38 ( 0.35 - 0.41 ) |
| Musculoskeletal And Connective Tissue Disorders | 154 | 0.26 ( 0.22 - 0.3 ) |
| Vascular Disorders | 146 | 0.7 ( 0.6 - 0.83 ) |
| Social Circumstances | 12 | 0.22 ( 0.12 - 0.39 ) |
| Surgical And Medical Procedures | 9 | 0.05 ( 0.03 - 0.1 ) |
| Endocrine Disorders | 55 | 1.82 ( 1.39 - 2.37 ) |
| Reproductive System And Breast Disorders | 19 | 0.28 ( 0.18 - 0.44 ) |
| Ear And Labyrinth Disorders | 7 | 0.15 ( 0.07 - 0.32 ) |
| Product Issues | 15 | 0.07 ( 0.04 - 0.11 ) |
| Congenital, Familial And Genetic Disorders | 2 | 0.07 ( 0.02 - 0.26 ) |

| **Supplementary Table S3**. Differences in clinical characteristics of serious and non-serious reports. | | | | | | | | | | | | | | |  |  |  |  |  |  |
| --- | --- | --- | --- | --- | --- | --- | --- | --- | --- | --- | --- | --- | --- | --- | --- | --- | --- | --- | --- | --- |
|  | | | | Serious cases | | | | Non-serious cases | | | p-value | | | |  |  |  |  |  |  |
|  | | | | n=1126 | | | | n=263 | | |  | | | |  |  |  |  |  |  |
| **SEX** | | | |  | | | |  | | |  | | | |  |  |  |  |  |  |
| F | | | | 265 (23.53%) | | | | 82 (31.18%) | | | ＜0.05 | | | |  |  |  |  |  |  |
| M | | | | 830 (73.71%) | | | | 170 (64.64%) | | |  | | | |  |  |  |  |  |  |
| Missing | | | | 31(2.75%) | | | | 11(4.18%) | | |  | | | |  |  |  |  |  |  |
| **WT** | | | |  | | | |  | | |  | | | |  |  |  |  |  |  |
| ＜80kg | | | | 217 | | | | 9 | | | ＜0.05 | | | |  |  |  |  |  |  |
| 80～100 kg | | | | 36 | | | | 4 | | |  | | | |  |  |  |  |  |  |
| ＞100 kg | | | | 31 | | | | 0 | | |  | | | |  |  |  |  |  |  |
| Missing | | | | 842 | | | | 250 | | |  | | | |  |  |  |  |  |  |
| **AGE** | | | |  | | | |  | | |  | | | |  |  |  |  |  |  |
| <18 | | | | 164 | | | | 19 | | | ＜0.05 | | | |  |  |  |  |  |  |
| 18～65 | | | | 169 | | | | 29 | | |  | | | |  |  |  |  |  |  |
| ＞65 | | | | 618 | | | | 67 | | |  | | | |  |  |  |  |  |  |
| Missing | | | | 175 | | | | 148 | | |  | | | |  |  |  |  |  |  |
| **Types of AEs** | | | |  | | | |  | | |  | | | |  |  |  |  |  |  |
| Alopecia | | | | 159（87.84%） | | | | 22(12.16%) | | | ＜0.05 | | | |  |  |  |  |  |  |
| Blister | | | | 37（75.51%） | | | | 12(24.49%) | | | ＞0.05 | | | |  |  |  |  |  |  |
| Cutaneous symptom | | | | 6（85.71%） | | | | 1(14.29%) | | | ＞0.05 | | | |  |  |  |  |  |  |
| Dermatitis | | | | 10（76.92%） | | | | 3(23.08%) | | | ＞0.05 | | | |  |  |  |  |  |  |
| Dermatitis allergic | | | | 4（66.67%） | | | | 2(33.33%) | | | ＞0.05 | | | |  |  |  |  |  |  |
| Drug eruption | | | | 42（93.33%） | | | | 3(6.67%) | | | ＞0.05 | | | |  |  |  |  |  |  |
| Dry skin | | | | 39（86.67%） | | | | 6(13.33%) | | | ＞0.05 | | | |  |  |  |  |  |  |
| Erythema | | | | 73（87.95%） | | | | 10(12.05%) | | | ＞0.05 | | | |  |  |  |  |  |  |
| Exfoliative rash | | | | 6（60.00%） | | | | 4(40.00%) | | | ＞0.05 | | | |  |  |  |  |  |  |
| Hyperkeratosis | | | | 1（50.00%） | | | | 1（50.00%） | | | ＞0.05 | | | |  |  |  |  |  |  |
| Leukoderma | | | | 3（75.00%） | | | | 1(25%) | | | ＞0.05 | | | |  |  |  |  |  |  |
| Lichenoid keratosis | | | | 6（85.71%） | | | | 1(14.29%) | | | ＞0.05 | | | |  |  |  |  |  |  |
| Nail disorder | | | | 1（50.00%） | | | | 1（50.00%） | | | ＞0.05 | | | |  |  |  |  |  |  |
| Palmar-Plantar erythrodysaesthesia syndrome | | | | 3（60.00%） | | | | 2(40%) | | | ＞0.05 | | | |  |  |  |  |  |  |
| Pruritus | | | | 186（85.71%） | | | | 31(14.29%) | | | ＞0.05 | | | |  |  |  |  |  |  |
| Psoriasis | | | | 4（66.67%） | | | | 2(33.33%) | | | ＞0.05 | | | |  |  |  |  |  |  |
| Rash | | | | 340（71.12%） | | | | 138(28.88%) | | | ＞0.05 | | | |  |  |  |  |  |  |
| Rash erythematous | | | | 26（68.42%） | | | | 12(31.58%) | | | ＜0.05 | | | |  |  |  |  |  |  |
| Rash macular | | | | 5（50.00%） | | | | 5（50.00%） | | | ＜0.05 | | | |  |  |  |  |  |  |
| Rash maculo-papular | | | | 24（72.73%） | | | | 9(27.27%) | | | ＞0.05 | | | |  |  |  |  |  |  |
| Rash papular | | | | 8（72.73%） | | | | 3(27.27%) | | | ＞0.05 | | | |  |  |  |  |  |  |
| Rash pruritic | | | | 26（66.67%） | | | | 13(33.33%) | | | ＞0.05 | | | |  |  |  |  |  |  |
| Rash vesicular | | | | 5（50.00%） | | | | 5（50.00%） | | | ＞0.05 | | | |  |  |  |  |  |  |
| Scab | | | | 2（66.67%） | | | | 1(33.33%) | | | ＞0.05 | | | |  |  |  |  |  |  |
| Sjs-Ten overlap | | | | 6（85.71%） | | | | 1(14.29%) | | | ＞0.05 | | | |  |  |  |  |  |  |
| Skin atrophy | | | | 1（50.00%） | | | | 1（50.00%） | | | ＞0.05 | | | |  |  |  |  |  |  |
| Skin burning sensation | | | | 5（83.33%） | | | | 1(16.67%) | | | ＞0.05 | | | |  |  |  |  |  |  |
| Skin discolouration | | | | 15（50.00%） | | | | 15（50.00%） | | | ＞0.05 | | | |  |  |  |  |  |  |
| Skin disorder | | | | 146（96.05%） | | | | 6(3.95%) | | | ＞0.05 | | | |  |  |  |  |  |  |
| Skin exfoliation | | | | 42（76.36%） | | | | 13(23.64%) | | | ＞0.05 | | | |  |  |  |  |  |  |
| Skin hyperpigmentation | | | | 3（37.50%） | | | | 5(62.50%) | | | ＜0.05 | | | |  |  |  |  |  |  |
| Skin irritation | | | | 2（66.67%） | | | | 1(33.33%) | | | ＞0.05 | | | |  |  |  |  |  |  |
| Skin lesion | | | | 8（80.00%） | | | | 2(20.00%) | | | ＞0.05 | | | |  |  |  |  |  |  |
| Skin reaction | | | | 24（64.86%） | | | | 13(35.14%) | | | ＜0.05 | | | |  |  |  |  |  |  |
| Skin toxicity | | | | 47（82.46%） | | | | 10(17.54%) | | | ＞0.05 | | | |  |  |  |  |  |  |
| Skin ulcer | | | | 7（63.64%） | | | | 4(36.36%) | | | ＞0.05 | | | |  |  |  |  |  |  |
| Stevens-Johnson syndrome | | | | 108（98.18%） | | | | 2(1.82%) | | | ＞0.05 | | | |  |  |  |  |  |  |
| Symmetrical drug-related intertriginous and flexural exanthema | | | | 6（66.67%） | | | | 3(33.33%) | | | ＞0.05 | | | |  |  |  |  |  |  |
| Toxic epidermal necrolysis | | | | 76（96.20%） | | | | 3(3.80%) | | | ＜0.05 | | | |  |  |  |  |  |  |
| Urticaria | | | | 6（85.71%） | | | | 1(14.29%) | | | ＞0.05 | | | |  |  |  |  |  |  |
| **Supplementary Table S4.** Clinical priority assessing results of disproportionality signals. | | | | | | | | | | | | | | | | | | |  | |
| PTs | | | | | | Reporting rate (cases/non-cases) | | | Signal stability (consistency across disproportionality analyses) | | | | | Reported case fatality rate (proportion of reports with death as outcome) | | IMEs/DMEs | | | | |
| Rash | | | | | | 478(04.45%) | | | 2 | | | | | 11.09% | | None | | | | |
| Pruritus | | | | | | 217(1.97%) | | | 2 | | | | | 13.82% | | None | | | | |
| Alopecia | | | | | | 181(1.64%) | | | 2 | | | | | 13.26% | | None | | | | |
| Skin disorder | | | | | | 152(1.37%) | | | 2 | | | | | 15.79% | | None | | | | |
| Stevens-Johnson syndrome | | | | | | 110(0.99%) | | | 2 | | | | | 45.45% | | DME | | | | |
| Erythema | | | | | | 83(0.74%) | | | 2 | | | | | 13.25% | | None | | | | |
| Toxic epidermal necrolysis | | | | | | 79(0.70%) | | | 2 | | | | | 74.68% | | DME | | | | |
| Skin toxicity | | | | | | 57(0.51%) | | | 2 | | | | | 12.28% | | None | | | | |
| Skin exfoliation | | | | | | 55(0.49%) | | | 2 | | | | | 14.55% | | None | | | | |
| Blister | | | | | | 49(0.44%) | | | 2 | | | | | 12.24% | | None | | | | |
| Drug eruption | | | | | | 45(0.40%) | | | 2 | | | | | 17.78% | | None | | | | |
| Dry skin | | | | | | 45(0.40%) | | | 1 | | | | | 8.89% | | None | | | | |
| Dermatitis bullous | | | | | | 40(0.36%) | | | 2 | | | | | 32.50% | | IME | | | | |
| Rash pruritic | | | | | | 39(0.35%) | | | 2 | | | | | 5.13% | | None | | | | |
| Rash erythematous | | | | | | 38(0.34%) | | | 2 | | | | | 5.26% | | None | | | | |
| Skin reaction | | | | | | 37(0.33%) | | | 2 | | | | | 16.22% | | None | | | | |
| Rash maculo-papular | | | | | | 33(0.29%) | | | 2 | | | | | 15.15% | | None | | | | |
| Skin discolouration | | | | | | 30(0.27%) | | | 2 | | | | | 6.67% | | None | | | | |
| Pigmentation disorder | | | | | | 17(0.15%) | | | 2 | | | | | 5.88% | | None | | | | |
| Toxic erythema of chemotherapy | | | | | | 14(0.12%) | | | 2 | | | | | 0.00% | | None | | | | |
| Dermatitis | | | | | | 13(0.12%) | | | 2 | | | | | 15.38% | | None | | | | |
| Rash papular | | | | | | 11(0.10%) | | | 2 | | | | | 9.09% | | None | | | | |
| Skin ulcer | | | | | | 11(0.10%) | | | 2 | | | | | 0.00% | | None | | | | |
| Exfoliative rash | | | | | | 10(0.09%) | | | 2 | | | | | 30.00% | | None | | | | |
| Rash vesicular | | | | | | 10(0.09%) | | | 2 | | | | | 0.00% | | None | | | | |
| Dermatitis exfoliative generalised | | | | | | 10(0.09%) | | | 2 | | | | | 10.00% | | DME | | | | |
| Skin lesion | | | | | | 10(0.09%) | | | 2 | | | | | 10.00% | | None | | | | |
| Symmetrical drug-related intertriginous and flexural exanthema | | | | | | 9(0.08%) | | | 2 | | | | | 11.11% | | None | | | | |
| Epidermal necrosis | | | | | | 8(0.07%) | | | 2 | | | | | 12.50% | | IME | | | | |
| Skin hyperpigmentation | | | | | | 8(0.07%) | | | 2 | | | | | 12.50% | | None | | | | |
| Skin erosion | | | | | | 8(0.07%) | | | 2 | | | | | 0.00% | | None | | | | |
| Pemphigoid | | | | | | 8(0.07%) | | | 2 | | | | | 25.00% | | IME | | | | |
| Toxic skin eruption | | | | | | 8(0.07%) | | | 2 | | | | | 25.00% | | IME | | | | |
| Erythema multiforme | | | | | | 7(0.06%) | | | 2 | | | | | 0.00% | | DME | | | | |
| Lichenoid keratosis | | | | | | 7(0.06%) | | | 2 | | | | | 0.00% | | None | | | | |
| Sjs-Ten overlap | | | | | | 7(0.06%) | | | 2 | | | | | 0.00% | | IME | | | | |
| Acute generalised exanthematous pustulosis | | | | | | 7(0.06%) | | | 2 | | | | | 0.00% | | IME | | | | |
| Cutaneous symptom | | | | | | 7(0.06%) | | | 2 | | | | | 14.29% | | None | | | | |
| Dermatitis exfoliative | | | | | | 6(0.05%) | | | 2 | | | | | 33.33% | | DME | | | | |
| Dermatitis allergic | | | | | | 6(0.05%) | | | 2 | | | | | 33.33% | | None | | | | |
| Decubitus ulcer | | | | | | 5(0.04%) | | | 2 | | | | | 20.00% | | None | | | | |
| Rash morbilliform | | | | | | 4(0.04%) | | | 2 | | | | | 25.00% | | None | | | | |
| Leukoderma | | | | | | 4(0.04%) | | | 2 | | | | | 25.00% | | None | | | | |
| Intertrigo | | | | | | 3(0.03%) | | | 2 | | | | | 0.00% | | None | | | | |
| DME, Designated medical event; IME, important medical event; PTs, preferred terms. | | | | | |  | | | | | |  | | | | |  | |  | |
| **Supplementary Table S5.** Time-to-onset analysis for signals with strong/moderate/weak prioritization. | | | | | | | | | | | | | | | | | | | |  |
| **Prioritization** |  |  |  | | **Weibull distribution** | | | | | | | | | | | | | **Failure type** | |  |
|  | **Cases** | **TTO (days)** | | | **Scale parameter** | | | | | **Shape parameter** | | | | | | | |  | |  |
|  | ***n*** | **Median (IQR)** | **Min–max** | | **α** | | **95% CI** | | | **β** | | | **95% CI** | | | | |  | |  |
| Strong | 40 | 13 (8.75–20) | 3–313 | | 23.71 | | 14.53–32.88 | | | 0.85 | | | 0.68–1.03 | | | | | Early failure | |  |
| Moderate | 534 | 11 (7–20) | 1–500 | | 22.43 | | 19.86–25 | | | 0.79 | | | 0.74–0.83 | | | | | Early failure | |  |
| Weak | 266 | 13 (7–22) | 1–314 | | 23.09 | | 19.72–26.47 | | | 0.87 | | | 0.8–0.95 | | | | | Early failure | |  |
| n, number of cases with available time-to-onset; IQR, interquartile range; TTO, Time-to-onset. When TTO is 0 days, the adverse event occurred within the same day with the therapy. | | | | | | | | | | | | | | | | | | | |  |
